# The Diagnostic Value of Examinations of the Spinal Fluid

**Published:** 1914-11

**Authors:** Allen H. Bunce

**Affiliations:** Atlanta, Ga.


### THE DIAGNOSTIC VALUE OF EXAMINATIONS OF THE SPINAL FLUID.

By Allen H. Bunce, A. B., M. D., Atlanta, Ga.

Since it is manifestly impossible for us to see the various pathological processes going on in the body during the course of any disease, we are forced to rely on our history, subjective symptoms, physical examination and laboratory examinations of the different body fluids and secretions in making our diagnoses. The last of these, that is, the laboratory examination, was the latest to be developed to such an extent that it could give us something definite with which to work. But, however well this may become perfected or however skillful it may be performed in any given case, a laboratory examination should never be allowed to take the place of, or supercede, a careful physical and clinical examination. These examinations should always be regarded merely as one of the aids in making a correct diagnosis. To be sure, they vary in value from those which are absolutely positive, as the finding of the tubercle bacillus in a suspected sputum, to those which are of a doubtful, and at best, only of a confirmatory nature.

The examination of the spinal fluid was one of the latest of the laboratory examinations to be perfected so that it now often gives absolutely positive data. And with our increasing knowledge of this very important fluid examinations of it will be performed much more frequently, and thereby often serve to establish a correct diagnosis in many otherwise doubtfhl cases.

The cerebro-spinal fluid is a secretion of the gland-like cells of the ependyma which covers the choroid plexus. It is contained within the space between the meninges and the brain and spinal cord which it completely envelopes. Normally, according to Howell, the amount of this fluid is 60 to 80 c. c. It is a clear, colorless, or slightly yellowish fluid having a specific gravity of 1005 to 1010 and contains from 0 to 6 cells per cubic millimeter. These cells are lymphocytes with an occasional endothelial cell. A substance is present which has the power of reducing Fehling's solution which is conceded to be dextrose by the best authorities. The proteids present are a mixture of globulin and proteose. Some peptone is found occasionally, also, urea is sometimes present. Serum albumin is very rarely present. The salts present are sodium and potassium oxide in small quantities.

To obtain this fluid the patient is placed on his side with the thighs flexed and shoulders bent forward, or the patient may be bent forward in the sitting position. In the middle of a line connecting the iliac crests a puncture is made with a trochar and cannula between the spinous processes of two of the lower lumbar vertabræ—usually the third and fourth. The fluid is allowed to flow out drop by drop until the required amount is obtained. About 5 c. c. is usually sufficient for a complete examination, but the amount withdrawn depends to some extent upon the provisional diagnosis and also upon the pressure. The reason for allowing the fluid to run slowly is to eliminate any shock from a too sudden lowering of the pressure. For instance, in cases of suspected brain tumor, although the fluid may be under very high pressure it should be removed extremely cautiously since sudden deaths have been reported following a too sudden removal of much fluid in this condition. As the fluid begins to flow if any blood should appear the first portion should be discarded as this is usually caused by the puncture of a small vein by the trochar and the presence of blood will interfere with the examination later. The pressure of the fluid may be taken while it is being withdrawn by connecting to the cannula a vertical glass tubing having a bore of one millimeter and being graduated in millimeters. The pressure may vary from 90 to 130 millimeters.

Of course, the examinations of the fluid to be made will depend to a great extent upon the provisional diagnosis. If the fresh fluid should be cloudy, a purulent meningitis is present and smears should be made from the fluid to determine the offending organism. If this cannot be determined from smears alone, cultures should be made, preferably on blood agar. The most common organisms causing a purulent meningitis are micrococcus intracellularis maningitidis, diplococcus pneumoniæ, bacillus influenzæ, and streptococcus pyogenes.

The diplococcus meningitidis of epidemic cerebrospinal meningitis, the most common of these, resembles the gonococcus in morphology, being a biscuit-shaped diplococcus which usually occurs within the pus cell. However, it may occur outside of the cells. It is Gram-negative and grows readily on all the common culture media.

In tuberculous meningitis the fluid is clear and under very high pressure—the pressure varying from 250 to 500 millimeters. If this fluid is allowed to stand for some time a thin pellicle or scum appears on the surface and extends down into the fluid. Smears made from this pellicle frequently show the tubercle bacillus. Another characteristic of tuberculous meningitis is a large increase in the lymphocytes which may number as high as several hundred per cubic millimeter.

Aside from the above forms of meningitis the most important application of an examination of the spinal fluid is in the diagnosis of cerebral lues, general paresis, and tabes dorsalis. In these diseases, as the result of a chronic posterior meningitis, there is usually a definite increase in the number of cells present. The increased number of cells here are of the lymphocytic variety.

For estimating the number of cells present the Fuchs—Rosenthal counting-chamber has been found to be the best on account of its larger ruled surface and greater depth. The ruled surface on this chamber measures 4 millimeters each way and the depth of the chamber is .2 of a millimeter, hence the cubic space represented here is 3.2 cubic millimeters. The counting is performed with the pipette for counting the white blood cells by drawing up to the .5 mark polychrome methylene blue and then filling to the mark 11 with the fluid. After mixing thoroughly a drop is placed in the counting chamber. All the lymphocytes and leukocytes are counted in all the squares and the result divided by 3 to get the number per cu. mm. 0 to 6 or 8 cells are considered normal, while if the count goes over 10 it is evident that we are dealing with a pathological fluid. This, at least, enables us to say that there is present an organic disease of the nervous system.

The increase in the globulins of the cerebrospinal fluid is best estimated by the "Phase I" of Nonne reaction. In this test the spinal fluid is allowed to run down the side of the tube containing at the bottom a saturated solution of ammonium sulphate. If the globulins are increased a white ring of contact will appear at the point where the two fluids meet. This increase may occur in all organic diseases of the central nervous system. It is present almost without exception in all syphilogenetic diseases of the brain and spinal cord. For this estimation of globulins the Butyric acid test of Noguchi may also be used.

Plant and Wassermann were the first to demonstrate the presence of a positive Wassermann reaction in the spinal fluid of patients suffering with tabes and general paresis. Later this test was found to be positive in the fluid of cerebrospinal syphilis. In determining whether or not the spinal fluid gives a positive Wassermann the tests should be done not only by using .2 cc of the fluid, but also with increasing quantities from .3 c. c. up to 1 c. c. of the fluid. It has been shown that this quantitative Wassermann will prove positive in syphilogenetic diseases as tabes, cerebral, spinal, and cerebrospinal syphilis and general paresis, while organic diseases of the nervous system which are not syphilitic as multiple sclerosis, cerebral and spinal tumors, never show the reaction, even when 1 c. c. of the fluid is used in making the test.

Finally, by a mastery of these methods of examinations of the spinal fluid and by their practical application we have an excellent aid toward the diagnosis of affections of the central nervous system, especially of the syphilitic nervous diseases.

824 Healey Building, Atlanta, Ga.

